# Self-powered bifunctional sensor based on tribotronic planar graphene transistors

**DOI:** 10.1038/s41598-021-01011-0

**Published:** 2021-11-02

**Authors:** Yanfang Meng, Guoyun Gao, Jiaxue Zhu

**Affiliations:** 1 State Key Laboratory of Advanced Optical Communications System and Networks, School of Electronics Engineering and Computer Science, Peking University, Beijing, 100871 China; 2 Center for Flexible Electronics Technology, Tsinghua University, Beijing, 100084 China; 3 School of Physics, University of Hong Kong, Zhuangyueming Physical Building, Hong Kong, 999077 China; 4 Key Laboratory of Microelectronic Devices and Integrated Technology, Institute of Microelectronics of the Chinese Academy of Sciences, Beitucheng West Road, Beijing, 100029 China

**Keywords:** Electronic devices, Sensors and biosensors

## Abstract

With the development of material science, micro-nano-fabrication and microelectronics, the higher level requirements are posed on the electronic skins (E-skin). The lower energy consumption and multiple functions are the imperative requirements to spurred scientists and mechanists to make joint efforts to meet. To achieve lower energy consumption, a promising energy-harvesting style of triboelectric nanogenerators (TENG) is incorporated into the field effect transistors (FETs) to play the important role for sensor. For bifunctional sensor, to harness the difficult for reflecting the magnitude of frequency, we resorted to synaptic transistors to achieve more intelligentization. Furthermore, with regards to the configuration of FET, we continued previous work: using the electrolyte gate dielectrics of FET—ion gel as the electrification layer to achieve high efficient, compact and extensively adoption for mechanosensation. The working principle of the GFET was based on the coupling effects of the FET and the TENG. This newly emerged self-powered sensor would offer a new platform for lower power consumption sensor for human–machine interface and intelligent robot.

## Introduction

Electronic skins (E-skin)^1^ emerged as electronic devices and electronic systems with powerful multi-functions of in-time sensing, storing data, tailoring, feedback, have significantly promoted the progress and reformation of society. The developments of material science, microelectronics, and integrated circuits have prompted the strong push towards flexible electronics dramatically^2–4^, achieving breakthrough in high sensitivity, responsiveness, high stretch ability, integration and intelligentialization. To suppress energy consumption, self-powered sensor has become a hot-pot, driving extensive attentions practically and academically. Triboelectrical nanogenerators (TENG), as an economical and high-efficient way to convert mechanical energy into electricity through coupling of tribo-electrification and electrostatic induction, have been driven extensive interests^5–10^. The TENG-based self-powered flexible electronic sensors have offer facile platform for monitoring temperature, humidity, physiological activity and other physical parameters^6,11–14^. To achieve high precision and high level of integration, the triboelectric potential originated form TENG can be incorporated into the field effect transistors (FETs) to tailor the transport properties of semiconductor channel for a myriad of versatile self-powered tribotronic devices^15–17^.The tribotronic devices have widespread applications in phototransistor, logic device, memristor, phototransistor, photovoltaic device and so on^18–20^.

Our previous work presented a noncontact mechanosensation active matrix based on tribotronic planar graphene transistors array to low-energy-cost real-time sensor and 2D imaging^21^. Apart from energy-efficiency, emulating the human’s skin of perception multiple stimuli is also an important issue to be addressed. For example, Sun et al.^22^ incorporated humidity sensor of GO, temperature of rGO into the pressure sensor (graphene electrode as electrode) to realize triple sensor for humidity, temperature and pressure. It is well-known that skin can percept both high frequent and lower frequent stimuli^23^. However, it was difficult to detect magnitude of frequency of the high frequent stimuli intuitively. To harness this problem, we resorted to plasticity behaviour synaptic transistor (transistor served both synaptic transistor and sensing unit). In synaptic transistor, synaptic weight (the increment phenomenon of later post-synaptic current compared with former counterpart) varied with the variation of frequency of pre-synaptic stimuli (signal)^24^. In our work, we assigned wind as stimuli source (the wind stimuli was utilized wind driving friction of electrofrictional layer of TENG), the increment of post-synaptic current of No. n pulse compared with No. n-1 pulse was explored to indicate the real-time frequency of wind that the sensor experienced. The mechanism involved triboelectric and electrostatic inducing. The work principal of magnetic field sensor was that (magnetic film and ion gel as friction layer) the magnetic field changed the distance between magnetic film and ion gel, giving rise to different signal. (Our previous work verified that the output of tribopotential-powered GFET had certain functional relationship with the triboelectric distance)^21^.

Incorporating synaptic transistors into E-skin has already been studied. Ravinder reported a printed synaptic transistor to create E-skin with positive mind and opened up a novel reformation: inserting Neuro layer into E-skin, possessing own Neuro and can immediately respond to external stimuli in absence of instruction. This project would invoke the reformation of wearable technology, flexible electronic and new generation computer^25^. Furthermore, graphene was selected as channel semiconductor for high mobility (chemical vapour deposited (CVD) graphene with mobility approaching 200,000 cm^2^ V^−1^ s^−1^ for the carrier density below 5 × 10^9^ cm^−2^ at a low temperature^26^), high transparency (the white light absorbance of a suspended graphene monolayer is 2.3% (or transmittance of 97.7%) with a negligible reflectance of < 0.1%^27^), excellent thermal stability and conductivity (3000–5000 W m^−1^ K^−1^)^28^, high Young’s modulus (~ 1 TPa)^29^ and large theoretical specific surface area (SSA, 2630 m^2^ g^−1^)^30^. Owing to these particular merits, graphene field effect transistor (GFET) applied as synaptic has reports^31^.

To summarize, we, developed a self-powered dual mode sensor for monitoring wind stimuli and magnetic stimuli, respectively. The wind stimuli represent the high frequency stimuli. One of the high frequent stimuli in daily life is physiotherapy instrument which acts quickly on the human body. We detected magnetic signals due to that magnetic fields or information related to magnetic fields exist in many places of our daily life. Particularly, we induced synaptic transistor into high frequency sensor to broad the horizon of the intelligentization of E-skin.

## Result and discussion

### The basic structure of the sensing system

**Figure 1:**
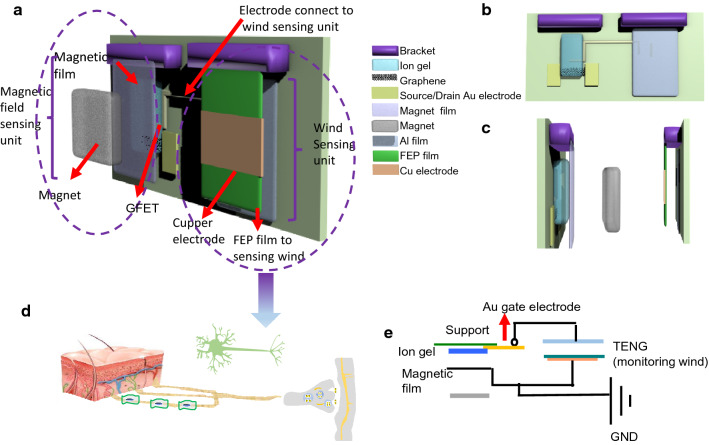
(**a**) Illustration of Self-powered bifunctional responsive sensor Based on Tribotronic Planar Graphene Transistors Schematically. Inset is the schematic illustration of the synaptic transistor to represent the work-style of sensor analogy to syanptic. (**b**) Front view of the device. (**c**) Lateral view of the device. (**d**) A schematic of the synapse. (**e**) Circuit diagram of the tribotronic GFET sensor.

Figure 1a presents a schematic illustration of the sensing device. The sensor contains two parts: the magnetic sensing unit and the wind sensing unit. With respect to the magnetic sensing unit, graphene field effect transistor (GFET) fixes vertically on frame. Facing to the GFET, a magnet is vertically installed on the sliding block of linear motor. The magnetic film vertically suspends between the GFET and magnet. (The magnetic film adheres onto electrode which grounded). With respect to wind sensing unit, the gate electrode of graphene transistor connects to an Al film of the TENG which adopt a contact-separation Cu/FEP/Al style (2 cm × 2 cm). The TENG composed of FEP film moving layer (0.5 mm thickness, connect to Cu electrode which grounded) and Al electrode (simultaneously as electrode and frictional layer and connected to Au gate electrode of FET). The wind stimuli drive FEP film and Al electrode contact and separation. Figure 1b presents the front view of device. The configuration of GFET follows our previous work: the ion gel is utilized as the electrolyte gate dielectric of FET as the electrification layer to achieve high efficient, compact and extensively adoption for mechanosensation. The width and length of graphene channel are 50 µm and 300 µm, respectively, with a 100 µm distance between gate and channel. The Au gate electrode of the GFET connects to the wind sensing unit via contacting to Al film. Figure 1c left panel and right panel display the left profile and right profile of the device, respectively. Figure 1d schematically illustrates the biological synaptic for the wind sensing unit as the sensor device can emulate. Figure 1e presents a circuit diagram of the sensor device. The wind induces the moving of frictional layer: the EFP film of TENG as pre-synaptic, serving as gate of transistor, tailors the transport of transistor and gives rise to variation output current as post-synaptic current. The mechanism of the device refers to the triboelectric and electrostaic inducing. Here, FEP film is selected as frictional material because of its thin and toughness to be qualified for producing the high frequency stimuli. Initially, FEP film and Al electrode adhered closely. According to the triboelectric series^32^, electrons transfer from the Al to FEP, leaving net positive electrostatic charges on the surface of Al layer and negative charges on the FEP film. Here, the charges with opposite polarities are totally balanced. Upon the wind flowing, the negatively charged FEP layer separates from the Al, the surplus positive charges on the Al film attract electrons of Au gate electrode of FET and change the charge distribution of the surface of ion gel, inducing an EDL at the interface between Au gate electrode of FET and ion gel, ion gel and graphene, leaving anions at ion-gel/graphene interface, which is amount to applying a negative voltage on the GFET. Consequently, the Fermi level of graphene channel is shifted downwards and the charge injection barrier between source electrode and graphene channel decreases, leading to an increment in the output current.

Figure S1 displays the Raman spectra of the graphene with two characteristic peaks at ~ 1597 cm^−1^ (G band) and at ~ 2694 cm^−1^ (2D band). The full width at half-maximum (~ 29 cm^−1^) of symmetric 2D band and the 2D/G intensity ratio (~ 2.5) are the solid evidences for testifying the monolayer graphene. Specially, G-band of graphene undergoes a blue shift from 1578 cm^−1^ to 1597 cm^−1^ suggests a slight p-type doping of graphene by moisture and oxygen^31^. With regards to intuitively reflect frequency of stimuli via synaptic plasticity of GFET, the stable and reversible triboelectric properties of GFET were essential. For desirable triboelectric properties, the stable electrical properties were the prerequisite.

### The triboelectrical properties of GFET

**Figure 2:**
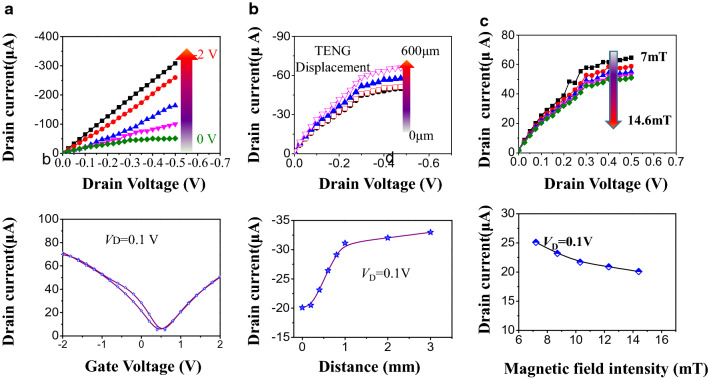
Electrical and magnetic performance of the GFET sensor. (**a**) Output characteristic (top bottom) and corresponding transfer curve (bottom panel) of GFET. (**b**) Output characteristic (top bottom) and corresponding transfer curve (bottom panel) of tribotronic GFET. (**c**) Output characteristic (top bottom) and corresponding transfer curve (bottom panel) GFET under varied magnitude of magnet field intensity.

Figure 2a top panel and bottom panel display the typical output characteristics and transfer curve of GFET under an applied gate voltage, respectively. It is clearly shown in Fig. 2a top panel that drain current (*I*_D_) increases from 51 μA to 308 μA with gate voltage (*V*_G_) increasing from 0 to 2 V at a drain voltage (*V*_D_) of 0.5 V. Figure 2a bottom panel obviously shows that the *I*_D_ increases with *V*_G_ increment in both positive and negative directions, indicating ambipolar charge transport property of graphene. The GFET can operate at a low gate voltage (< 2 V) due to the ultrahigh capacitance of the ion gel gate dielectric (6 ~ 7 μF cm^−2^). Figure 2b top panel and 2b bottom panel illustrate the typical output triboelectric characteristics and triboelectric transfer curve of GFET with different distance between FEP film and Al film, respectively. With respect to TENG-driving at a given *V*_D_, the absolute value of *I*_D_ increased (51 μA to 67.1 μA) as the distance between FEP film and Al film of TENG increases from 0 to 600 µm, which is equivalent to applying increased gate voltage in negative direction. With respect to corresponding tribotronic transfer curve (at a given *V*_D_ of 0.1 V), when the contact distance increased from 0 to 1 mm, the absolute value of *I*_D_ increases from 20.1 μA to 32.99 μA, which is equivalent to applying a gate voltage of 0.15 V. Initially, the FEP film and Al film are in full contact with each other, resulting in a charge neutralization state. On basis of the triboelectric series^32^, electrons transfer from the Al film to FEP film, leaving positive charges on the surface of Al film and negative charges on the surface of FEP film. By now, the charges with opposite polarities achieve complete equilibrium and the Fermi level of graphene not changes. Upon the negatively charged FEP film vertically separates from the Al at a distance away, positive charges on the surface of Al attracted the electronic of Au electrode and produced surplus positive charge on surface of Au electrode. Consequently, induced EDL is produced within ion gel, leaving negative ions at ion-gel/graphene interface, which is amount to applying a negative pulse voltage on the semiconductor channel. Therefore, the Fermi level of graphene channel is shifted downwards, giving rise to an increment of the output current. To elucidate the dynamic tribotronic performance through external TENG, real-time triboelectrical tests were performed, as shown in Fig. S2. The variation of *I*_D_ was stable, verifying the reliability of triboelectric-driving GFET for synaptic device.

The magnetic field stimuli behaviors were also investigated (Fig. 2c top panel: output curve under varied magnitude of magnetic field, bottom panel: transfer curve under varied magnitude of magnetic field). *I*_D_ decreases (64.6 μA to 51 μA) as the magnitude of magnet field intensity increases from 7 mT to 14.6 mT, equivalent to applying increased gate voltage in positive direction. With respect to corresponding tribotronic transfer curve (at a given *V*_D_ of 0.1 V), when the magnitude of magnet field intensity increases from 7 mT to 14.6 mT, the *I*_D_ decreases from 25.1 μA to 20.1 μA. The variation rate was 0.66 µA mT^−1^ (As the magnitude of magnet field greater than 7 mT). It is mentioned that due to overcoming the weight of magnetic film, only the magnet field greater than 7 mT can drive the magnetic film separated from ion gel. (As the magnitude of magnet field less than 7 mT, the *I*_D_ was no unchanged). As a results, the functional relationship of *I*_D_ and magnitude of magnet field is *I*_D_ = *I*_0_ − 2.69% *I*_0_ (H-7) (Here, *H* is referred as the current magnitude of magnet field), *I*_0_ is assigned to the initial value of drain current. As the ion gel is intrinsically ion liquid gelation with the cross-linked polymer, its coupling with triboelectric field in different directions will lead to the distribution of electric double layers (EDLs) in opposite directions. According to the triboelectric series^32^, the triboelectric series ion gel can be utilized as a neutral friction material. (Our previous work has pioneeringly adopted ion gel as both the dielectric layer of GFET and friction layer of TENG. As a result, the triboelectric potential was directly coupled to GFET^22^). Compared with ion gel, PDMS is a negative triboelectric material. In absence of magnetic field, positive charges and negative charges are left on the surface of ion gel and PDMS, respectively, originating from the tribo-electrification and electrostatic induction between ion gel and PDMS. Owing to equivalent positive charge and negative charge were inter-neutralization, there is no change in ion gel and graphene. As magnet approach nearly to magnet film, due to attract interaction, the negatively charged magnet film leave away from ion gel, the anions in ion gel are attracted to compensate the accumulated positive charges, while the cations migrate to the ion-gel/graphene interface and the Fermi level of graphene is up-sifted. On the contrary, when the magnet leaves away from the magnet film, the negatively charged magnet conformally adhered the ion gel again, the positive charge on the surface of the ion gel and negative charge on surface of the magnet film were inter-neutralization again and there is no EDL on ion-gel/graphene interface and Fermi level of graphene restored. Furthermore, the magnitude of magnetic field has closely related to the distance of contact-and-separation between magnetic film and ion gel and determines the triboelectric potential posed on GFET, further posing impact on output current of GFET.

### The plasticity behavior of GFET

**Figure 3:**
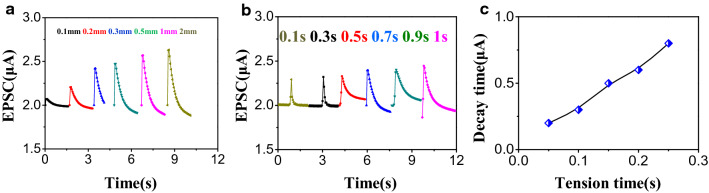
(**a**) Single-pulse behavior of triboelectric graphene synaptic device under different triboelectric distance. (**b**) Single-pulse behavior of triboelectric graphene synaptic device under different tension time. (**c**) Decay time of triboelectric graphene synaptic device under different tension time of single-pulse behavior.

The synaptic weight is represented by the degree of connectivity between the pre- and post-neurons is simply described by the postsynaptic current (PSC) magnitude when the input spikes stimulate the synapse. The ability of controlling and retaining the synaptic weight over time is defined as synaptic plasticity, classifying into two forms: short-term plasticity (STP) and long-term plasticity^33–35^. The synaptic behaviors were extensively investigated. The single pulse behaviors were firstly explored. It is well-known that synaptic behaviors are closely related to the intensity and tension time. As shown in Fig. 3a, the electrical post-synaptic current (EPSC) increases as the distance between FEP and Al of the TENG increased (same tension time), implying the stronger stimuli, the stronger responsiveness received. (The extracted curves of the EPSC-distance with tribopotential as pre-synaptic stimuli are shown in Fig. S3 left panel). With respect to tension time, as shown in Fig. 3b, under the given triboelectric distance of 10 μm, the EPSC increases as tension (spiking duration) time increases. This spike-timing-dependent plasticity (STDP) phenomenon is maybe explained by that the longer tension time brought about more charge accumulation in ion gel, posing stronger control on channel of GFET. (The extracted curves of the EPSC-tension time with tribopotential as pre-synaptic stimuli shows in Fig. S3 right panel). Figure 3c presents decay time^36^ and the memory retention of the synapses is in agreement with the commonly used forgetting curve y = b × t − m, where y, t, b and m represent memory retention, time, the fitting constant for scaling, and the power function rate, respectively.

**Figure 4:**
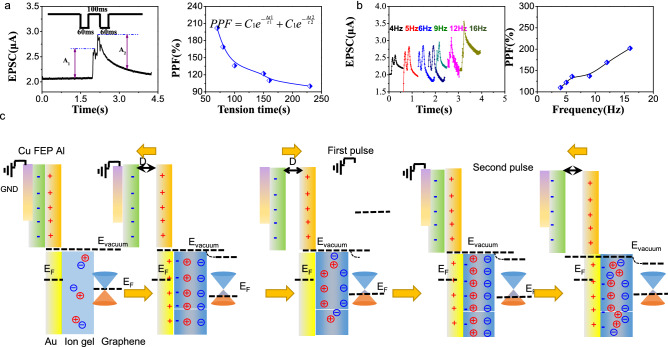
(**a**) PPF behavior of triboelectric graphene synaptic device under sequent-dual-pulse stimuli (triboelectric distance = 200 μm, spike time interval Δtpre = 60 ms, spike tension time = 10 ms) left panel: EPSC of device under sequent double-pulse stimuli. Right panel: PPF of device under varied tension time of sequent double-pulse stimulis. (**b**) The EPSC of device under varied frequency of dual-spike pulse wind serve as triboelectric pulse (4 Hz–16 Hz) (left panel). The corresponding extracted frequency-dependent PPF (right panel). (**c**) The mechanism for PPF behaviour.

Based on single pulse behavior, the PPF behavior of device is extensively explored. Synaptic plasticity can also be modified by the time intervals when two sequential spikes stimulate the synapse. Such behavior has been considered the paired-pulse facilitation (PPF) feature of a synapse, providing flexibility for several neuronal tasks, such as simple learning and information processing in the frequency domain^37^. Figure 4a left panel displays the EPSC triggered by a pair of tribopotential (triboelectric distance = 200 μm) spike with an inter-spike interval (Δt_pre_ = 60 ms, spike duration time = 100 ms). Owing to PPF (= (A_1_ − A_2_)/A_1_ × 100%) as a function of the time interval when two sequential spikes generate PSC peaks, Fig. 4a right panel displays the PPF-Δt_pre_ curve values exponentially decay as the interval increases, and this decay characteristic showed the best agreement with the fitting function with two decaying time constants of *C*_1_exp (− Δ*t*_1_/τ_1_) + *C*_2_exp(− Δ*t*_2_/*τ*_2_). Using *C*_1_ and *C*_2_ as fitting parameters, we obtained two decaying time constants,*τ*_1_ = 16 ms and *τ*_2_ = 2741 ms. This PPF behavior is similar to the reported one in biological synapses, indicating our synaptic transistor can functionally mimic the biological synapse. As analogous to single pulse, the dual and multiple was also heavily related to the magnitude of stimuli intensity and tension time. Variation frequency of dual-spike pulse wind serve as triboelectric pulse (4 Hz–16 Hz), the EPSC was measured (Fig. 4b left panel). It can be clearly observed that higher frequency brought about the more remarkable increment. The corresponding extracted frequency-dependent PPF is shown in Fig. 4b right panel.

The frequency dependent synaptic was also studied using 10 pulses with different frequencies (5 Hz–12.5 Hz) and the EPSC was measured (Fig. S4a). The corresponding extracted frequency-dependent A_2_/A_1_ is showed in Fig. S4b. It was basically the same as the relationship of PPF in Fig. 4b. The A_N_/A_1_ as the frequency at 5 Hz and is shown in Figure. S4c, suggesting that the ESPC increased as increasing the number of pulse spikes.

Figure 4c illustrated the mechanism of PPF behaviour of sensor. At the initial position, the Al film and FEP film were in full contact with each other, resulting in a charge neutralization state. According to the triboelectric series, electrons transferrsed from the Al film to FEP film, leaving net positive electrostatic charges on the surface of Al layer and negative electrostatic charges on the surface of FEP. At this stage, the produced triboelectric charge with opposite polarities were fully balanced with no influence on the Fermi level of graphene. Once the negatively charged FEP layer was vertically separated from the Al at a certain distance, positive charges on the surface of Al attracted the electrons of Au electrode and produce surplus positive charge on surface of Au electrode. As a result, the EDL appears within the ion gel, leaving negative ions at ion-gel/graphene interface, which is equivalent to applying a negative pulse voltage on the synaptic device. As a result, the Fermi level of graphene channel is shifted downwards, leading to an increase of the drain current. As the FEP generally contacts with Al again, positive electrostatic charge on the surface of Al layer and negative electrostatic charges on the surface of FEP achieve balance again. However, due to the intrinsical delay relaxation characteristic of ion gel^38,39^, when the pulse interval is shorter than relaxation time of ions within ion gel, some of these trapped ions are reluctant to return to their equilibrium positions before the second spike initiated. Consequently, these residual ions induce additional charges in the channel, leading to an increase in channel current and PPF behavior. When the FEP separates from Al again, positive charges on the surface of Al attract the electrons of Au electrode, giving rise to surplus positive charge on surface of Au electrode and producing EDL within ion gel. This newly emerged EDL pluses the remanent regular-aligned charges at ion-gel/grapheme interface during the first pulse process, increasing negative ions at ion-gel/graphene interface, which explained for that increment of second ESPC compared to ESPC of first pulse (PPF behavior). Furthermore, the magnitude of the PPF has closely related to the pulse interval. Large pulse interval allowed these mobile ions to relax back to their equilibrium positions prior to the second spike application leading to weak channel current modulation and lower PPF index.

### The real-time sensing of bifunctional sensor

**Figure 5:**
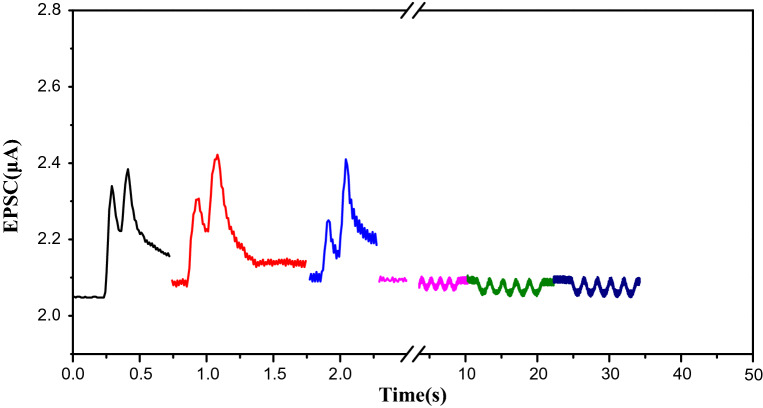
(a) Bifunctional sensing signals (left part: high frequency of wind stimuli, right part: low frequency of magnetic field stimuli) of triboelectric GFET.

On account of impractical of simultaneously controlling multiple units for magnetic field, we implemented only one transistor to sensor. Further work will conduct for the active matrix E-skin for multiple-functional sensor needs. If wind stimuli and magnetic stimuli conduct simultaneously, two signals will overlap and it is difficult to distinct between them. So wind and magnetic stimuli were carried out successively. As shown in Fig. 5, for wind stimuli sensing part, the synaptic plasticity of dual stimuli are 115%, 157% and 200%, respectively, calculated from 4b, the frequency-dependent PPF. (Frequency of 4 Hz, 5 Hz, 6 Hz, 9 Hz, 12 Hz and 16 Hz are corresponded to PPF of 110%, 122%, 136%, 137%, 169%, 200%, respectively). It can be estimated that the frequency of wind stimuli are between 4 Hz–5 Hz, between 6 Hz–9 Hz and 16 Hz. For magnetic field stimuli sensing part, the increasing percentage of output current are 1.428%, 2.14% and 2.38%, as the magnitude of magnetic field intensity varies. According to Fig 3c, as magnitude of magnetic field intensity increases from 7.2 mT to 14.6 mT, *I*_D_ decreases from 25.1 µA to 20.1 µA (*V*_D_ = 0.1 V), with the variation rate 0.66 µA mT^−1^. The every 1mT magnitude of magnetic field intensity induces current varied 2.63% (the calculated magnitude of magnetic field intensity is according to percentage rather than the absolute decrease value).The extracted functional relationship of *I*_D_ and magnitude of magnet field is *I*_D_ = *I*_0_ − 2.63% *I*_0_ (H-7). So, the calculated magnitudes of magnetic field intensity are 7.53 mT, 7.81 mT and 7.90 mT. Therefore, this sensor shows promising applications for intuitively monitoring frequency by means of synaptic transistor, as well as sensing the magnitude of magnetic field intensity to realize dual sensing.

## Conclusions

As a summarization, to emulate the multiple stimuli-responsiveness of skin, this work explored high- frequent stimuli wind and low-frequent stimuli magnet bifunctional responsiveness of the GFET sensor. Particularly, we proposed a strategic method to overcome the obstacle of reflecting the magnitude of frequency of high-frequent stimuli in transitional E-skin. Inspired by PPF phenomenon (the increment behavior of No. n pulse compared with No.n-1 pulse) of synaptic transistor, we achieved indicating the frequency of high-frequency wind stimuli with perceptual intuition by measurement of synaptic weight of the EPSC (referred to No. n pulse compared with No.n-1 pulse). The mechanism involved triboelectric and electrostatic inducing. The wind stimuli is by means of wind driving friction of electrofrictional layer of TENG. The work principal of magnetic field sensor is that (magnetic film and ion gel as friction layer) the magnetic field changes the distance between magnetic film and ion gel and produces to different signals. We ingeniously incorporated synaptic transistor into E-skin, opening a new avenue for intelligentization of E-skin.

## Methods

### Materials and methods

High quality mono-layer graphene films were prepared on a Cu foil (thickness 25 μm, 99.8%) via CVD process:

#### CVD—deposition graphene

Initially, a Cu foil (25 μm) was cleaned with a mixture solution containing 98wt% H_2_SO_4_ and 30 wt% H_2_O_2_ for 15 min. Subsequently, Cu foil was rinsed with DI water and dried under a nitrogen flow. Inserting the Cu foil into a quartz tube was prior to pumping air in the chamber. Upon the pressure in the quartz tube reaching 5 × 10-3 Torr, the Cu foil was annealed at 1000 °C for 30 min, under a continuous flowing H_2_ (10 sccm) atmosphere. 5 sccm CH_4_ was then introduced for graphene growth. After 30 min, the CH_4_ flow was ceased, while the H2 flow was continued to apply until the tube was cooled to room temperature. Thus, large area (10 × 10 cm^2^) mono-layer graphene was obtained on a Cu foil^40,41^.

#### Fabrication of magnetic film

PDMS and curing agent mixed according to the ratio of 10:1, adding to 5–10 wt% magnetic powder (size 500 nm–2 μm), fully mixed and pouring on glass slide (which pre- treated using trichlorosilicane), spining coating at 500–800 rpm for 1 min. Cured 2 h at 80 °C and peeled off the film from the glass slide.

#### Device fabrication

PET substrate (120 μm) was cleaned under sonication with acetone, isopropanol and DI water, respectively. Transfer graphene included the following steps: oxygen plasma etching (200 W, 7 s) of the graphene layers on the backside of Cu foil; poly(methyl methacrylate) (PMMA) supporting layer was spin-coated at 4000 rpms for 5 s onto the graphene patterns on the Cu foil; the Cu foil was electrochemically etched using an ammonium persulfate aqueous solution (20 g L^−1^); the floated monolayer graphene films was then transferred onto the PET substrate; the supporting PMMA layer was removed by dipping into the hot acetone (~ 60 °C ) for 5 min. Graphene was then patterned by standard photolithography (AZ 5214 as photoresist, exposure for 10 s under UV light of 275 W) and subsequent oxygen plasma etching (20sccm O2, 150 W, 5 s) process. Residual photoresist was removed with acetone. The ion gel paste-shape liquid composed of the 1-ethyl-3-methylimidazolium bis(trifluoromethylsulfonyl)imide ([EMIM][TFSI]) ion liquid, the poly(ethylene glycol) diacrylate (PEGDA) monomer, and the 2-hydroxy-2methylpropiophenone (HOMPP) photo-initiator (weight ratio of 90:7:3) was dipped onto the patterned graphene. Under UV exposure, polymerization of PEGDA was initiated by the reaction between monomers and radicals originated from HOMPP to produce cross-linked structure. The unexposed region was washed away using DI water. The graphene region in contact with the ion gel served as the active channel, whereas the other region functioned as the source/drain electrodes^42,43^.

The as-prepared magnetic film was fixed on the frame, vertically hanging at front of ion gel of GFET.

The utilized TENG was based on a contact-separation mode between FEP film (connect to Gu electrode which ground) and Al film. Al film connected to gate electrode of FET.

### Characterization

UV–Vis measurements were carried out with an ultraviolet spectrophotometer (HOPBA). The electrical and tribotronic performance of the GFETs were implemented with an Agilent B1500A Semiconductor Device analyzer. During the tribotronic performance test, a displacement motor driven by a computer controlled stepping motor was connected to a probe station. All measurements were conducted under ambient condition^19,21^.

## Supplementary Information

Supplementary Information.
