# Supplementary material for: Self-powered bifunctional sensor based on tribotronic planar graphene transistors
Source: Sci Rep. 2021 Nov 2;11:21483. doi: 10.1038/s41598-021-01011-0 (PMC8563961; doi:10.1038/s41598-021-01011-0)
Supplement: Supplementary file 1 — Supplementary Information. [file 41598_2021_1011_MOESM1_ESM.docx]

Supporting information for

**Self-powered bifunctional sensor Based on Tribotronic Planar Graphene Transistors**

**Yanfang Meng*** 1,2,+**, Guoyun Gao** 3,+**, Jiaxue Zhu** 4**,**

1 State Key Laboratory of Advanced Optical Communications System and Networks, School of Electronics Engineering and Computer Science, Peking University,Beijing 100871, China.

2 Center for Flexible Electronics Technology, Tsinghua University, Beijing, 100084, China.

3 School of Physics, University of Hong Kong, Zhuangyueming physical building, Hong Kong, 999077, China.

4 Key Laboratory of Microelectronic Devices and Integrated Technology, Institute of Microelectronics of the Chinese Academy of Sciences, Beitucheng West Road, Beijing 100029, China

* Corresponding author: [yanaimengmeng@126.com](mailto:yanaimengmeng@126.com)

**Supplementary Text**

**Fig. SI.1**. Raman spectrum of the pristine graphene by CVD grown monolayer.

**Fig. SI.2**. The real-time triboelectrical tests of tribotronic GFETs. (*V*_DS_=0.5V, distance=1mm).

**Fig. SI.3.** Left panel: The extracted curves of post-synaptic current (EPSC)-triboelectric distance with tribopotential as pre-synaptic stimuli. Right panel: The extracted curves of post-synaptic current (EPSC)- triboelectric distance with tribopotential as pre-synaptic stimuli.

**Fig. SI.4** Left panel: EPSC of GFET under varied frequency of triboelectric pulse. Right panel: Extracted A_N_/A_1_Variation frequency of triboelectric pulse (N=1-10, 1Hz-10Hz) of EPSC(triboelectric distance=200μm,spike time interval Δtpre=ms, spike tension time =10ms)

.

**Supplementary Figures**

**Fig. SI.1.**Raman spectrum of the pristine graphene by CVD.

**Fig. S2.**The real-time triboelectrical tests of tribotronic GFETs (*V*_DS_=0.5V, distance=1mm).

**Fig. S3** Left panel: The extracted curves of post-synaptic current (EPSC)-triboelectric distance with tribopotential as pre-synaptic stimuli. Right panel: The extracted curves of post-synaptic current (EPSC)- triboelectric distance with tribopotential as pre-synaptic stimuli.

**Fig. S4** (a) EPSC of GFET under varied frequency of triboelectric pulse

(b) PPF of the second pulse of 10 pulses. (c)Extracted A_N_/A_1_ of 5Hz spike pulse of EPSC(triboelectric distance=200μm,spike time interval Δtpre=ms, spike tension time =10ms).
